# Supplementary figures and images for: Brazilian and Mexican propolis and their possible mechanism of action against non-enveloped viruses
Source: PLoS One. 2025 May 13;20(5):e0323129. doi: 10.1371/journal.pone.0323129 (PMC12074604; doi:10.1371/journal.pone.0323129)

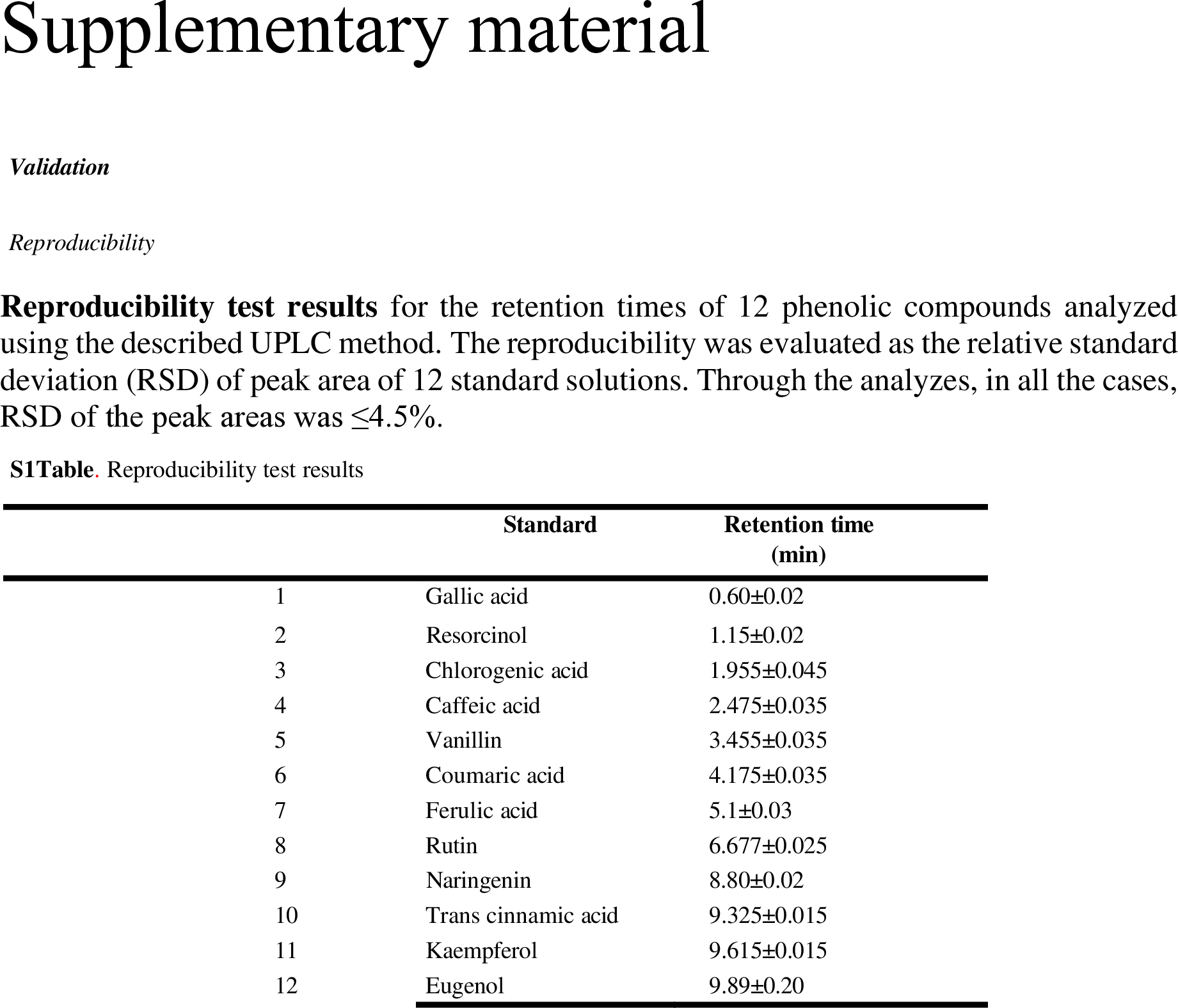

Supplement: S1 Table — For the retention times of 12 phenolic compounds analyzed using the described UPLC method. (TIF) [file pone.0323129.s001.tif]

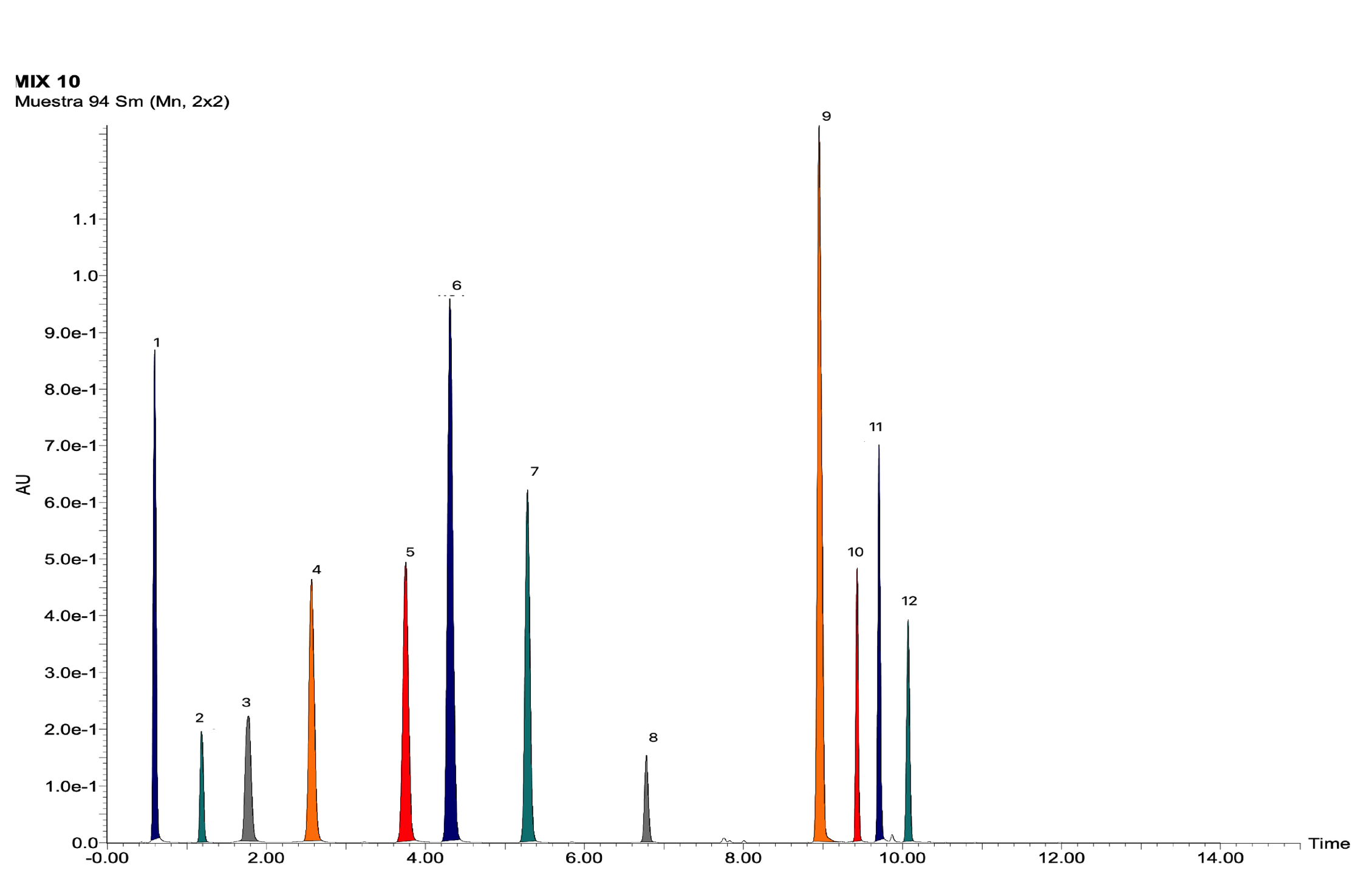

Supplement: S1 Fig — Peaks: 1, gallic acid; 2, resorcinol; 3, chlorogenic acid; 4, caffeic acid; 5, vanillin; 6, coumaric acid; 7, ferulic acid; 8, rutin; 9, naringenin; 10, quercetin;11, kaempferol, and 12, eugenol. (TIF) [file pone.0323129.s002.tif]

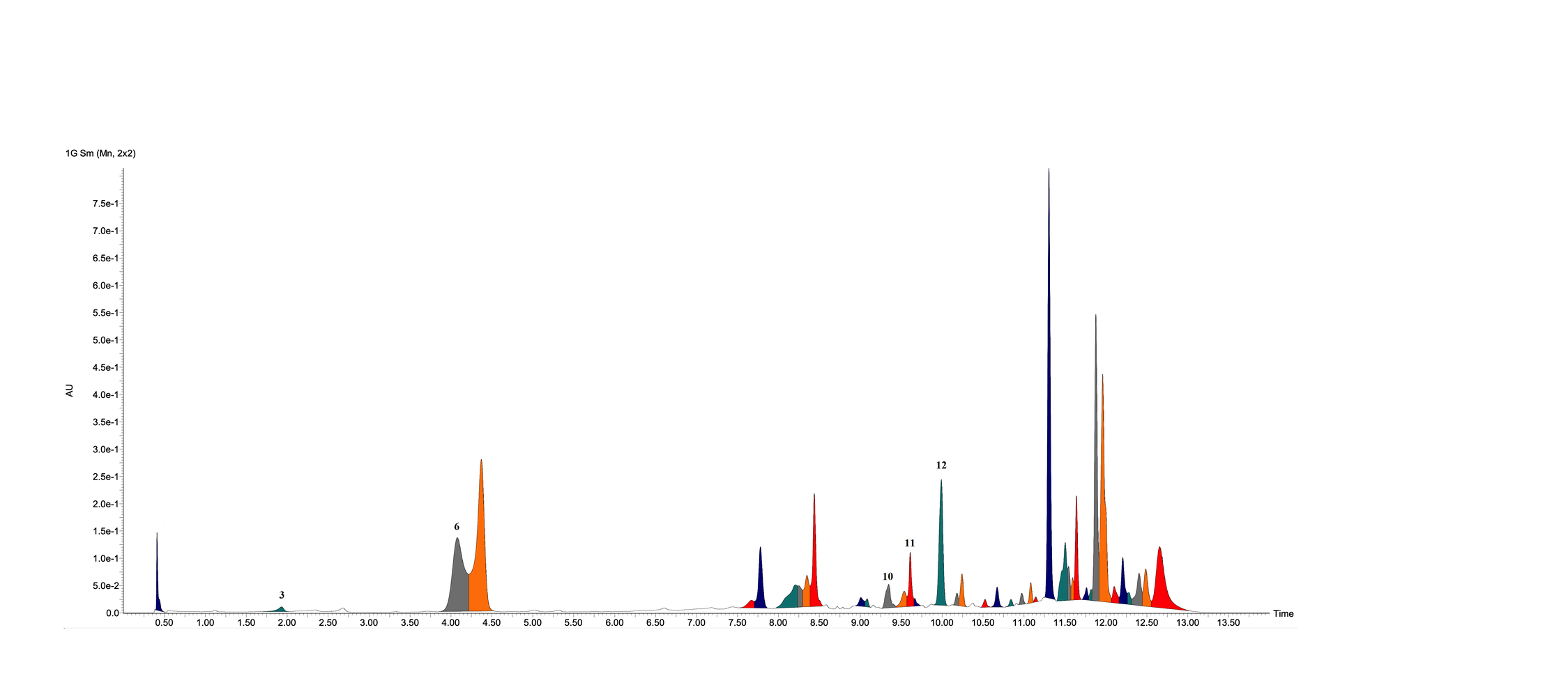

Supplement: S2 Fig — Peaks: (3) naringenin, (6) coumaric acid, (10) quercetin, (11) kaempferol, (12) eugenol. (TIF) [file pone.0323129.s003.tif]

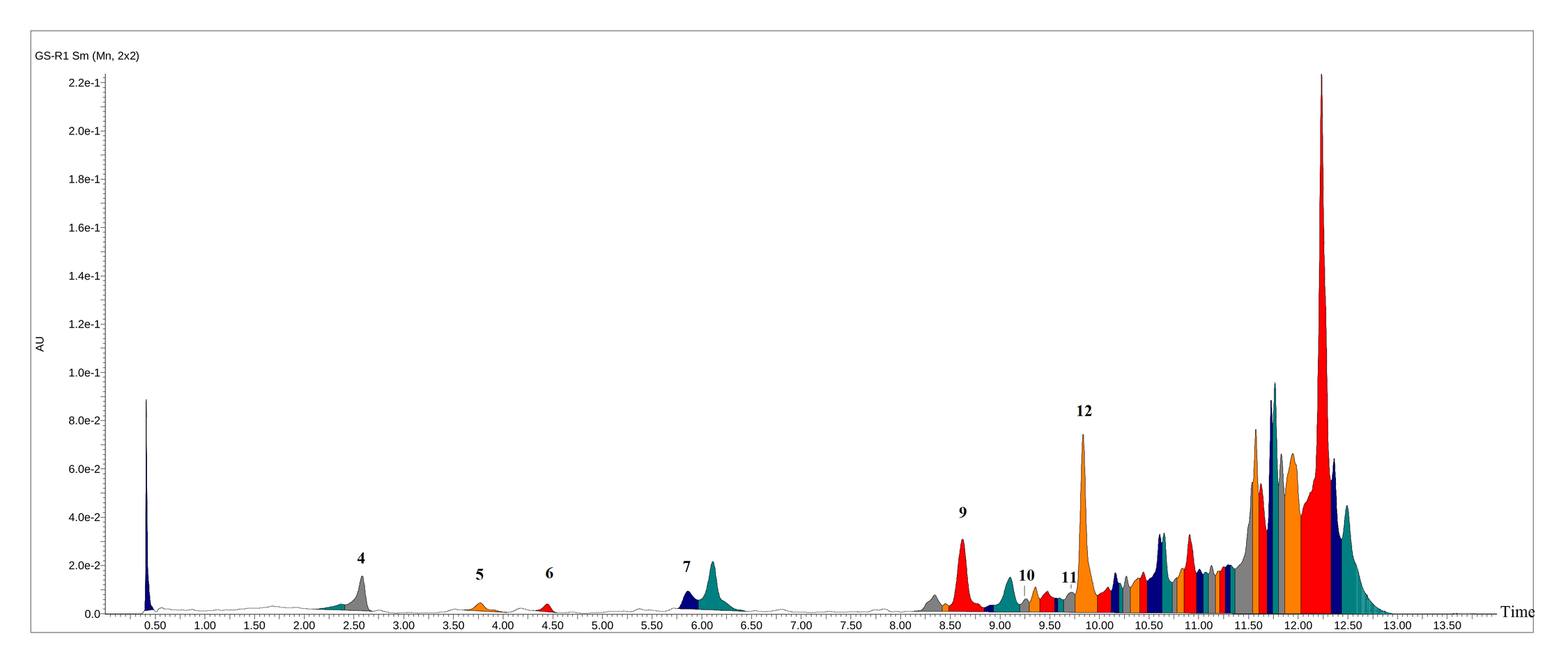

Supplement: S3 Fig — (9) naringenin, (10) quercetin, (11) kaempferol, (12) eugenol. (TIF) [file pone.0323129.s004.tif]

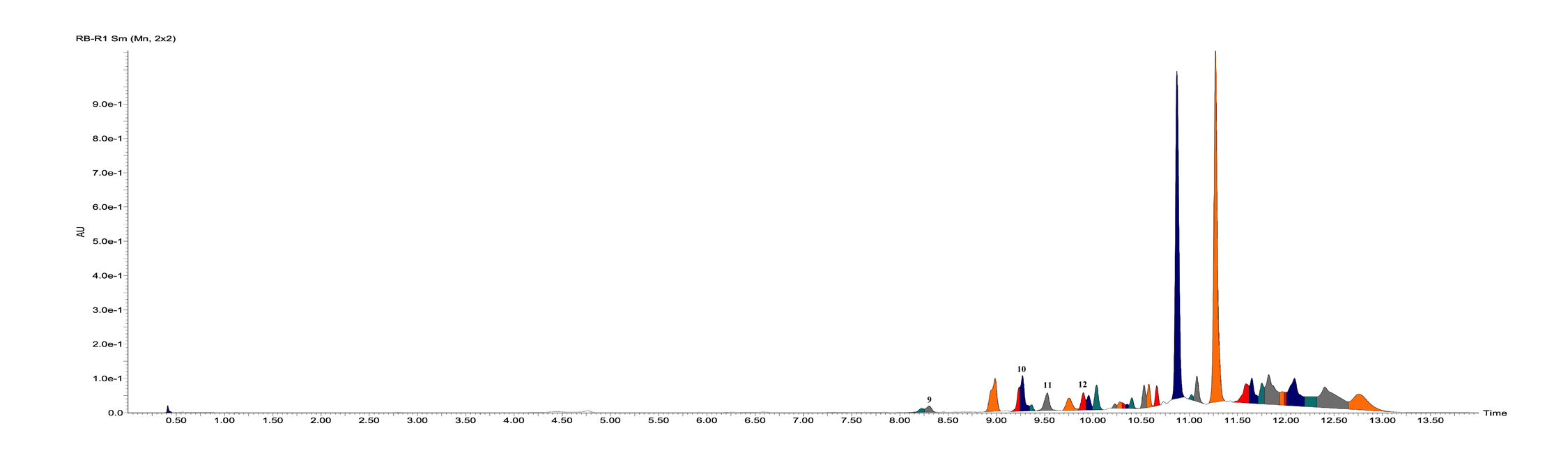

Supplement: S4 Fig — (3) naringenin, (4) caffeic acid, (5) vanillin, (6) coumaric acid, (7) ferulic acid, (9) naringenin, (10) quercetin, (11) kaempferol, (12) eugenol. (TIF) [file pone.0323129.s005.tif]

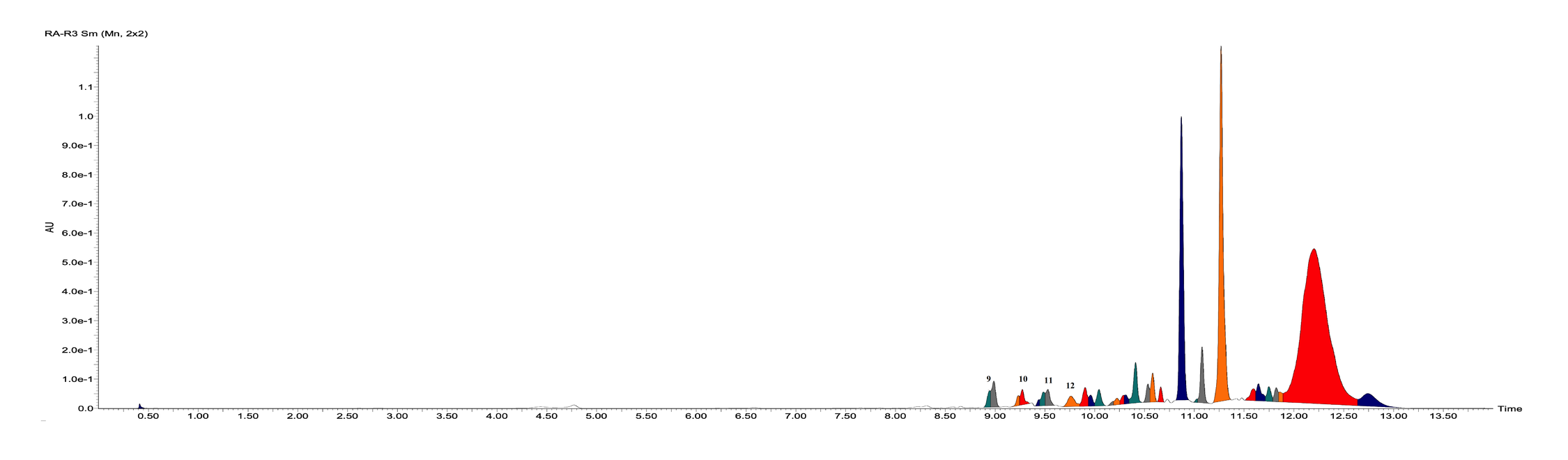

Supplement: S5 Fig — (9) naringenin, (10) quercetin, (11) kaempferol, (12) eugenol. (TIF) [file pone.0323129.s006.tif]
