# Supplementary material for: Brazilian and Mexican propolis and their possible mechanism of action against non-enveloped viruses
Source: PLoS One. 2025 May 13;20(5):e0323129. doi: 10.1371/journal.pone.0323129 (PMC12074604; doi:10.1371/journal.pone.0323129)
Supplement: Attachment — Submitted filename: Response to Reviwers.docx [file pone.0323129.s009.docx]

**'Response to Reviewers'**

**Reviewers' comments**

Has the statistical analysis been performed appropriately and rigorously?

Reviewer #1: No

Reviewer #2: I Don't Know

The statistical analysis has been conducted appropriately and rigorously. In the methodology section, particularly in the description of the in-silico study, additional lines were included to enhance clarity and understanding. These added explanations can now be found in lines (146-148) of the manuscript.

**Reviewer #1:** The submitted manuscript is interesting for the approached subject. I have some concerns although, and before I may recommend the Editorial office a possible publication, I am asking the authors to clarify this. Page 4, line 97: the authors stated, "until constant weight". If the propolis extract is dried completely, how is this statement used? I believe the extract is completely dried and then weighted to make afterwards the dilutions requested for the study. Am I right? (maybe I misunderstood).

The authors would like to thank the reviewer for their observation. You are correct in your interpretation, and we apologize if the wording was not entirely clear. The phrase "until constant weight" refers to the drying process of the propolis extract until no further weight loss is observed, indicating that the extract has reached its stable weight. Indeed, after achieving constant weight, the extract is weighed and then diluted according to the study requirements. For further clarification, the method has been updated, now in lines (97-100).

Page 14, line 294-295: prenylated phenyl propanoids and isoflavonoids are not detected in the studied samples? They are the main flavonoids in red and green propolis as stated also in the ISO 24381:2024 - Bee propolis — Specifications... The authors did not find them at all? These compounds have a huge bioactivity for green and red propolis.

We appreciate your reference to **ISO 24381:2024,** which provides guidelines for the detection of chemical compounds in propolis. Following to this standard, our study identified several phenolic compounds, including phenylpropanoids or phenolic acids (caffeic acid, ferulic acid, and p-coumaric acid), flavonoids and derivates (Rutin, quercetin, naringenin, kaempferol) phenols (resorcinol, eugenol, vanillin), phenolic esters such as chlorogenic acid, and Gallic acid were identified, as detailed in (Table 2).

However, the absence of certain characteristic compounds, such as artipellin C (prenylated phenylpropanoids), formononetin (isoflavone), or pinocembrin, does not necessarily imply their non-existence in the analyzed samples. Our approach focused on identifying compounds that, based on the literature and a previous review published by our team (Silva-Beltrán et al., 2021), as well as our prior experience, were expected be present in both types of propolis (from Brazil and Mexico), given the diversity of ecosystems in each region. Therefore, we did not specifically target these markers but instead focused on compounds previously reported in propolis samples from both countries. In this regard, the validity of the in-silico study was ensured by using only properly characterized molecules, which guaranteed the reliability of the computational models employed.

To further address your concern, we have included an additional clarification in the discussion section, which can be found in lines [283-292]. We would also to mention that our research team plans to continue this study by incorporating other compounds considered markers for different types of propolis exploring a broader range of bioactive effects and mechanisms of action.

From the chromatograms presented as supplementary files, I could not figure out what were the identified compounds ...
Please clear this matter..

We apologize for any confusion. Compound identification was based on retention times and absorption spectra obtained during chromatographic analysis, using external standards. For clarity, we included legible figures to better highlight the compounds identified in the chromatograms.

**S1 Fig.** **UPLC chromatogram of phenolic compound standards analyzed at 280 nm [y axis = intensity (absorbance unit, AU); x axis = retention time (min).** Peaks: 1, gallic acid; 2, resorcinol; 3, chlorogenic acid; 4, caffeic acid; 5, vanillin; 6, coumaric acid; 7, ferulic acid; 8, rutin; 9, naringenin; 10, quercetin;11, kaempferol, and 12, eugenol.

**S2 Fig.** **UPLC chromatogram of phenolic compounds present in G**. Peaks: (3) naringenin, (6) coumaric acid, (10) quercetin, (11) kaempferol, (12) eugenol.

**S3 Fig**. **UPLC chromatogram of phenolic compounds present in GS**. (9) naringenin, (10) quercetin, (11) kaempferol, (12) eugenol.

**S4 Fig**. **UPLC chromatogram of phenolic compounds present in RB**. (3) naringenin, (4) caffeic acid, (5) vanillin, (6) coumaric acid, (7) ferulic acid, (9) naringenin, (10) quercetin, (11) kaempferol, (12) eugenol.

**S5 Fig**. **UPLC chromatogram of phenolic compounds present in RA.** (9) naringenin, (10) quercetin, (11) kaempferol, (12) eugenol.

**References**

Silva-Beltrán, N.P.; Umsza-Guez, M.A.; Ramos Rodrigues, D.M.; Gálvez-Ruiz, J.C.; de Paula Castro, T.L.; Balderrama-Carmona, A.P. **Comparison of** the Biological Potential and **Chemical Composition of Brazilian and Mexican Propolis**. Appl. Sci. **2021**, 11, 11417. <https://doi.org/10.3390/app112311417>

Reviewer #2: Analysis of chemical composition can be improved. I consider that there are some errors or omissions that must be addressed:

Thank you for your comments. We appreciate your suggestions and have carefully reviewed your analysis. Below are the responses to each of them.

1. I don't see how the quantification process was done. UV detector or mass detector? Mass spectrometer conditions are not declared

We apologize for any confusion. To clarify, the quantification process was carried out using a **UV detector**, not a mass spectrometer. The quantification was based on **retention times** and **absorption spectra** obtained during chromatographic analysis, using external standards. The use of external standards allowed for accurate compound quantification through calibration curves. We have corrected the description of the protocol for greater clarity. Please refer to the updated details in line (126).

2. Did authors employ distilled water for ULPC-UV-MS analysis?

We employed **HPLC-grade solvents**, which is standard practice to ensure the quality and accuracy of the chromatographic analysis. For further clarification, the method has been updated. Please see now the update in line (129).

3. How the compounds were identified? Mass data? Only retention time? Propolis is a very complex mixture of compounds in which both isomers and isobars are common. Comparis on based on retention times is not enough!!!!

We appreciate your comment and understand the complexity of the propolis samples, which contain isomers and isobars. We would like to clarify that the identification of the compounds was not based solely on retention times, but on a combination of two methods:

Retention Times: External standards were used to establish retention times, as shown in Figure S1, allowing for an initial identification of the compounds.

Absorption Spectra: Identification was corroborated by comparing the absorption spectra obtained during chromatographic analysis, providing greater certainty. This protocol was previously validated by our team (Balderrama-Carmona et al., 2020), where reproducibility, linearity, and detection limits were reported, along with the absorption spectra of the identified compounds. For clarity, enlarged figures have been included in the supplementary files, in order to better highlight the compounds identified in the chromatograms.

Balderrama-Carmona, A.P.; Silva-Beltrán, N.P.; Gálvez-Ruiz, J.-C.; Ruíz-Cruz, S.; Chaidez-Quiroz, C.; Morán-Palacio, E.F. Antiviral, Antioxidant, and Antihemolytic Effect of *Annona muricata* L. Leaves Extracts. Plants **2020**, 9, 1650. https://doi.org/10.3390/plants9121650

5. I don’t see **typical chemical constituents** of both red and green Brazilian propolis in table 2

We acknowledge that the absence of certain characteristic or typical compounds in Brazilian propolis, such as artipellin C, formononetin, does not necessarily imply their non-existence in the analyzed samples. Our approach focused on identifying compounds that, based on the literature and a previous review published by our team (Silva-Beltrán et al., 2021), as well as our prior experience, we knew would be present in both types of propolis (from Brazil and Mexico), given the diversity of ecosystems in each region. In this regard, the validity of the in-silico study was also ensured by maintaining a more rigorous and precise approach, using only properly characterized molecules, which ensured the reliability of the computational models used. However, to address your concern, we have decided to include an additional clarification in the discussion section, which can be found in line [283-292].

Furthermore, we would like to mention that our research team plans to continue this study by incorporating other compounds considered **markers or typical chemical** constituents for different types of propolis, to explore a broader range of bioactive effects and mechanisms of action.

Silva-Beltrán, N.P.; Umsza-Guez, M.A.; Ramos Rodrigues, D.M.; Gálvez-Ruiz, J.C.; de Paula Castro, T.L.; Balderrama-Carmona, A.P. **Comparison of** the Biological Potential and **Chemical Composition of Brazilian and Mexican Propolis**. Appl. Sci. 2021, 11, 11417. <https://doi.org/10.3390/app112311417>
