# Supplementary material for: Brazilian and Mexican propolis and their possible mechanism of action against non-enveloped viruses
Source: PLoS One. 2025 May 13;20(5):e0323129. doi: 10.1371/journal.pone.0323129 (PMC12074604; doi:10.1371/journal.pone.0323129)
Supplement: Attachment — Submitted filename: Review Report PLOS ONE.docx [file pone.0323129.s008.docx]

**Brazilian and Mexican Propolis and their possible Mechanism of Action against nonenveloped Viruses**

The subject is vital and the manuscript is well structured. I have some suggestions to improve the quality of the manuscript.

**The title**

Mechanism of action of an antibiotic is not just its binding to a target protein, It involves the subsequent actions on specific processes such as translation (protein synthesis) transcription (RNA synthesis), replication (DNA synthesis) and reverse transcription (Synthesis of DNA from RNA) [1-3]. I suggest the following title:

Brazilian and Mexican Propolis and their *Invitro* and *Insilco* activities against nonenveloped Viruses

**Abstract**

1- Rephrase the abstract to reflect the suggested title
2- you stated (Propolis samples were characterized by ultra-performance liquid chromatography (UPLC) before testing), I suggest to rephrase it stating that you measured the concentration of 12 phenolic compounds using the UPLC.

**Introduction**

I suggest to add a paragraph about the non-envelope viruses; their types, severity and structure

**Material and methods:**

Please describe in details how you measured the 12 phenolic acids. How you created the standard curves and what are the quality parameters you used to control your assays.

**Results**

Add the standard curves of the phenolic acids including the straight line equation, R^2^ and r values.

**Discussion**

Discuss the results of the 12 phenolic acids you investigated

**References**

1. Eshboev F, Mamadalieva N, Nazarov PA, Hussain H, Katanaev V, Egamberdieva D, Azimova S. Antimicrobial Action Mechanisms of Natural Compounds Isolated from Endophytic Microorganisms. Antibiotics. 2024; 13(3):271. <https://doi.org/10.3390/antibiotics13030271>.
2. Ye L, Zhang J, Xiao W, Liu S. Efficacy and mechanism of actions of natural antimicrobial drugs. Pharmacology & Therapeutics. 2020; 216: 107671. <https://doi.org/10.1016/j.pharmthera.2020.107671>.
3. Neu HC, Gootz TD. Antimicrobial Chemotherapy. In: Baron S, editor. Medical Microbiology. 4th edition. Galveston (TX): University of Texas Medical Branch at Galveston; 1996. Chapter 11. Available from: <https://www.ncbi.nlm.nih.gov/books/NBK7986/>
