# Supplementary material for: Brazilian and Mexican propolis and their possible mechanism of action against non-enveloped viruses
Source: PLoS One. 2025 May 13;20(5):e0323129. doi: 10.1371/journal.pone.0323129 (PMC12074604; doi:10.1371/journal.pone.0323129)
Supplement: Attachment — Submitted filename: Response to reviewers .docx [file pone.0323129.s010.docx]

**'Response to Reviewers'**

**Reviewer #1**: I am satisfied with the answers that the authors gave to my concerns. For this reason I will suggest the editorial office to take into consideration for publication the study you have been conducted

Thank you for your positive feedback and for taking the time to review our manuscript. We truly appreciate your thoughtful comments and your support in considering our study for publication.

**Reviewer #2**:

On line 125 the phrase ¨distilled water¨ still remains

We have reviewed now is line 140.

I consider that the retention time and the comparison with the standards are not sufficient to unequivocally determine the structure of a compound in a product as complex as propolis. Nowadays, characterization by mass spectrometry is common and even several multiple reactions monitoring transitions are required to identify a compound.

The current requirements of validation processes for HPLC-MS constitute evidence of the rigor of structural characterization. Otherwise, there is a risk of errors in structural identification.

We sincerely appreciate your valuable comments and your interest in enhancing the quality of our work. We fully acknowledge your concerns regarding the unequivocal identification of compounds in a complex matrix such as propolis. However, we would like to emphasize that our methodological approach is based on validated techniques, which have been extensively reported in the literature for the identification and quantification of compounds in complex natural products like propolis. For instance, Devequi et al. (1) successfully used HPLC-UV for the analysis of propolis, and Zhang et al. (2023) (2) demonstrated that both HPLC-UV and HPLC-MS/MS can be effectively used for the analysis of propolis when good quality control conditions.

Our analytical protocol has been rigorously validated in terms of reproducibility (RSD < 5%), linearity (R² > 0.99), and detection limits, ensuring the reliability of the reported results. The information was added (see Lines 134-138; 168-178) and **S1 Table**.

Additionally, the compounds identified were selected based on their previously documented occurrence in propolis from Brazil and Mexico (1–6), minimizing the risk of misidentification.

Considering these points, we are confident that the data obtained remain scientifically robust and provide relevant insights into the composition of the analyzed samples. We have also made sure to transparently describe our methodology, providing sufficient detail for readers to understand both the scope and the limitations of our approach.

We recognize that techniques such as mass spectrometry would add significant value to future studies. Therefore, we will take your recommendation into careful consideration for the next stage of our study, which will focus on working with propolis markers. To this end, we plan to conduct the research using HPLC-MS.

Once again, we sincerely appreciate your constructive feedback, which will undoubtedly contribute to the ongoing improvement of our research.

**Lines 134-138**

The phenolic compounds were quantified using external standard curves prepared from pure standards. For compound identification, the absorption spectrum generated by integrating the area under the curve at the retention time detected in the standards was compared. Quality parameters were reproducibility, linearity, and limit of quantification (LOQ).

**Lines:168-178**

A phenolic profile was performed on samples G, GS, RA, and RB using 12 standards. Standard curves for the phenolic compounds are shown in **S2 figure**, while chromatograms and detection peaks for G, GS, RB, and RA are provided in the supplementary material (**S3, S4, S5, and S6 Figs**). Quality control parameters included linearity (R² > 0.99) and precision (RSD < 4.5%), as detailed in **S1 Table**. Linearity was evaluated by analyzing different standard solutions, and calibration curves were constructed by plotting the under-curve area versus concentration. The curves exhibited slopes of 86.136, intercepts of -105.74, and correlation coefficients of 0.99, with a limit of quantification (LOQ) of 0.2 μg/mL. A total of 10 constituents were identified, and **Table 2** shows the concentration of the phenolic components detected in the red propolis from Brazil, as well as in the green propolis from Brazil and Mexico (Table 2).

References

1. Devequi-Nunes D, Machado BAS, Barreto GdA, Rebouças Silva J, da Silva DF, da Rocha JLC, et al. Chemical characterization and biological activity of six different extracts of propolis through conventional methods and supercritical extraction. PLoS One. 2018;13(12):e0207676
2. Zhang Y, Cao C, Yang Z, Jia G, Liu X, Li X, et al. Simultaneous determination of 20 phenolic compounds in propolis by HPLC-UV and HPLC-MS/MS. Journal of Food Composition and Analysis. 2023;115:104877
3. Hernandez J, Goycoolea FM, Quintero J, Acosta A, Castañeda M, Dominguez Z, et al. Sonoran propolis: chemical composition and antiproliferative activity on cancer cell lines. Planta medica. 2007;73(14):1469-74.
4. Silva-Beltrán NP, Umsza-Guez MA, Ramos Rodrigues DM, Gálvez-Ruiz JC, de Paula Castro TL, Balderrama-Carmona AP. Comparison of the biological potential and chemical composition of Brazilian and Mexican propolis. Applied Sciences. 2021;11(23):11417.
5. Devequi-Nunes D, Machado BAS, Barreto GdA, Rebouças Silva J, da Silva DF, da Rocha JLC, et al. Chemical characterization and biological activity of six different extracts of propolis through conventional methods and supercritical extraction. PLoS One. 2018;13(12):e0207676.
6. Salatino A, Salatino MLF, Negri G. How diverse is the chemistry and plant origin of Brazilian propolis? Apidologie. 2021:1-23.

**Reviewer #3**: The manuscript needs some corrections to improve its quality. I attached a file containing my comments

**The title**

Mechanism of action of an antibiotic is not just its binding to a target protein, It involves the subsequent actions on specific processes such as translation (protein synthesis) transcription (RNA synthesis), replication (DNA synthesis) and reverse transcription (Synthesis of DNA from RNA) [1-3]. I suggest the following title:

Brazilian and Mexican Propolis and their *Invitro* and *Insilco* activities against nonenveloped Viruses

We appreciate your comments and, in line with your observations, we have had the opportunity to improve our results and identify more relevant data derived from the mechanisms of action, which led us to decide not to change the title. With the intention of strengthening it (title), we have made improvements to the abstract (30-32; 34-37), discussion (361-374), and conclusion (380-383), where we have added information related to the mechanism of action.

**Discussion**

Lines 361-374

Furthermore, **Figure 1A** shows that phenolic compounds exhibit greater affinity for DNA bacteriophages. This finding is consistent with the in vitro study (Table 3), which indicates that the DNA bacteriophage ΦX174 is more sensitive.

To link the in vitro effects with the known functions of specific viral proteins, we selected four proteins involved in capsid structure, genome binding, and infection mechanisms. Specifically, we analyzed MS2 (PDB ID: 1AQ3) and ΦX174 (PDB ID: 2BPA) both of which are required for genome attachment-dependent capsid assembly.

To determine the effect on viral processes, we selected spike proteins, including 5TC1 (chain M) for MS2 and 1CD3 (chain G) for ΦX174. The former is responsible for virus attachment to the F pilus and genome delivery, while the latter is associated with effective infection (1)

Therefore, the antiviral properties of propolis G and GS observed in this study could be attributed to their higher content and composition of phenolic compounds, which enhance synergistic (protein-ligand) effects, facilitating interactions that neutralize the capsid proteins required for genome attachment-dependent capsid assembly of both non-enveloped viruses.

**Conclusion**

Lines 380-383

Altogether, our results highlight the effect of propolis on phage infection and suggest a possible mechanism of action through binding to capsid proteins, which could prevent the proper formation of the capsid-genome complex and block viral infection.

**Abstract**

1- Rephrase the abstract to reflect the suggested title.

The information was added Lines: 30,32.

*In silico* molecular docking was also conducted to determine binding energy and molecular interaction and putative mechanism of propolis phenolic compounds with two viral capsid proteins and two proteins involved in viral replication and infection.

The information was added Lines: 34-37

Molecular docking simulations revealed that ΦX174 was also more sensitive to the phenolic compounds and that the combination of quercetin and kaempferol showed the greatest antiviral effect as a possible mechanism, through binding to the viral capsid proteins near the viral genome binding sites.

2- you stated (Propolis samples were characterized by ultra-performance liquid chromatography (UPLC) before testing), I suggest to rephrase it stating that you measured the concentration of 12 phenolic compounds using the UPLC.

The information was added: (lines 26-28)

Propolis samples were characterized by performing a phenolic profile using ultra-performance liquid chromatography (UPLC), which included 12 phenolic compounds such as phenylpropanoids, flavonoids, phenols, and phenolic acids

**Introduction**

I suggest to add a paragraph about the non-envelope viruses; their types, severity and structure

The information was added: (lines 65-71)

Several studies have employed bacteriophage models to investigate the in vitro virucidal effects of natural substances [4, 12]. Among them, the bacteriophages MS2 (single-stranded RNA) and ΦX174 (single-stranded DNA), both non-enveloped virus, have been employed as viral models. Non-enveloped viruses are typically more resistant to adverse environmental conditions and to the action of antimicrobials [13]. The MS2 bacteriophage has been used as an experimental surrogate for SARS-CoV-2 [14], while ΦX174 used as an enteric virus surrogate [15]

**Material and methods:**

Please describe in details how you measured the 12 phenolic acids How you created the standard curves and what are the quality parameters you used to control your assays.

The information was added: (lines 133-140)

The phenolic compounds were quantified using external standard curves prepared from pure standards, with concentrations ranging from 0.1 to 100 μg/mL. For compound identification, the absorption spectrum generated by integrating the area under the curve at the retention time detected in the standards was compared. Quality parameters were reproducibility, linearity, and limit of quantification (LOQ)

**Results**

Add the standard curves of the phenolic acids including the straight-line equation, R^2^ and r values.

The information was added: (lines 168-178)

**Phenolic composition of propolis**

A phenolic profile was performed on samples G, GS, RA, and RB using 12 standards. Standard curves for the phenolic compounds are shown in **S2 figure**, while chromatograms and detection peaks for G, GS, RB, and RA are provided in the supplementary material (**S3, S4, S5, and S6 Figs**). Quality control parameters included linearity (R² > 0.99) and precision (RSD < 4.5%), as detailed in **S1 Table**. Linearity was evaluated by analyzing different standard solutions, and calibration curves were constructed by plotting the under-curve area versus concentration. The curves exhibited slopes of 86.136, intercepts of -105.74, and correlation coefficients of 0.99, with a limit of quantification (LOQ) of 0.2 μg/mL.

A total of 10 constituents were identified, and **Table 2** shows the concentration of the phenolic components detected in the red propolis from Brazil, as well as in the green propolis from Brazil and Mexico (Table 2).

**Discussion**

Discuss the results of the 12 phenolic acids you investigated

The requested information has now been integrated, and you can view it in lines

Lines 292-299

Propolis extracts from Mexico and Brazil were analyzed to evaluate their antiviral activities in vitro and explore possible mechanisms using of action molecular docking on the detected chemical constituents. A targeted phenolic profile was performed on samples G, GS, RA, and RB, including phenylpropanoids or phenolic acids (caffeic acid, ferulic acid, and p-coumaric acid), flavonoids and their derivatives (rutin, quercetin, naringenin, kaempferol), phenols (resorcinol, eugenol, vanillin), and phenolic esters such as chlorogenic acid and gallic acid, as detailed in **Table 2**. The presence of these compounds was corroborated through chromatographic analysis.

Lines 306-323

However, this phenylpropanoid was only detected in GS. Brazilian green propolis is mostly composed of prenylated phenylpropanoids, mainly artepillin C; Brazilian red propolis mainly contains isoflavonoids (biochanins, medicarpin) [24]. Although we acknowledge the potential presence of other characteristic compounds in the studied propolis samples, this study focused on identifying compounds that, based on previous research conducted by our team, we knew could be detected in both Brazilian and Mexican propolis[9]. This approach ensured the validity of the **docking** analysis. Consequently, were also detected in Brazilian propolis compounds, such as chlorogenic acid, caffeic acid, vanillin, coumaric acid, ferulic acid, naringenin, kaempferol, eugenol and quercetin [9]. Quercetin, kaempferol and eugenol were detected in all propolis samples G, GS, RB, RA. In addition to the compound identified, it was observed that red propolis presents significant concentrations of kaempferol with 45.752, 73.86 and 38.47 mg/g for G, RB, RA respectively. However, rutin (quercetin 3-O-rutinoside) was not detected in all propolis samples, likely because the flavonoids in propolis are mostly aglycons, making rutin a rare major component. Another compound that was not detected was resorcinol, which was expected to be found in Brazilian propolis, particularly in samples from hives near mangroves in the states of Bahía (RB) and Alagoas (RA) (29). The absence of specific compounds in propolis depends on the botanical diversity of the region.

Lines: 361-363

Furthermore, **Figure 1A** shows that phenolic compounds exhibit greater affinity for DNA bacteriophages. This finding is consistent with the in vitro study (Table 3), which indicates that the DNA bacteriophage ΦX174 is more sensitive.

Lines 368-379

To link the in vitro effects with the known functions of specific viral proteins, we selected four proteins involved in capsid structure, genome binding, and infection mechanisms. Specifically, we analyzed MS2 (PDB ID: 1AQ3) and ΦX174 (PDB ID: 2BPA) both of which are required for genome attachment-dependent capsid assembly.

To determine the effect on viral processes, we selected spike proteins, including 5TC1 (chain M) for MS2 and 1CD3 (chain G) for ΦX174. The former is responsible for virus attachment to the F pilus and genome delivery, while the latter is associated with effective infection (1)

Therefore, the antiviral properties of propolis G and GS observed in this study could be attributed to their higher content and composition of phenolic compounds, which enhance synergistic (protein-ligand) effects, facilitating interactions that neutralize the capsid proteins required for genome attachment-dependent capsid assembly of both non-enveloped viruses.
